# GLP-1 Receptor Agonists and Related Mental Health Issues; Insights from a Range of Social Media Platforms Using a Mixed-Methods Approach

**DOI:** 10.3390/brainsci13111503

**Published:** 2023-10-24

**Authors:** Davide Arillotta, Giuseppe Floresta, Amira Guirguis, John Martin Corkery, Valeria Catalani, Giovanni Martinotti, Stefano L. Sensi, Fabrizio Schifano

**Affiliations:** 1 School of Clinical Pharmacology and Toxicology, University of Florence, 50121 Florence, Italy; davide.arillotta@yahoo.it; 2 Psychopharmacology, Drug Misuse and Novel Psychoactive Substances Research Unit, School of Life and Medical Sciences, University of Hertfordshire, Hatfield AL10 9AB, UK; giuseppe.floresta@unict.it (G.F.); amira.guirguis@swansea.ac.uk (A.G.); j.corkery@herts.ac.uk (J.M.C.); v.catalani@herts.ac.uk (V.C.); giovanni.martinotti@gmail.com (G.M.); 3 Department of Drug and Health Sciences, University of Catania, 95124 Catania, Italy; 4 Pharmacy, Swansea University Medical School, Faculty of Medicine, Health and Life Science, Swansea University, Swansea SA2 8PP, UK; 5 Department of Neurosciences, Imaging and Clinical Sciences, University of Chieti-Pescara, 66100 Chieti, Italy; stefano.sensi@unich.it; 6 Center for Advanced Studies and Technology (CAST), Institute of Advanced Biomedical Technology (ITAB), University of Chieti-Pescara, Via dei Vestini 21, 66100 Chieti, Italy

**Keywords:** GLP-1 receptor agonists, semaglutide, mental health, depression, anxiety, sleep disorders, food cravings, social media, netnography

## Abstract

The emergence of glucagon-like peptide-1 receptor agonists (GLP-1 RAs; semaglutide and others) now promises effective, non-invasive treatment of obesity for individuals with and without diabetes. Social media platforms’ users started promoting semaglutide/Ozempic as a weight-loss treatment, and the associated increase in demand has contributed to an ongoing worldwide shortage of the drug associated with levels of non-prescribed semaglutide intake. Furthermore, recent reports emphasized some GLP-1 RA-associated risks of triggering depression and suicidal thoughts. Consistent with the above, we aimed to assess the possible impact of GLP-1 RAs on mental health as being perceived and discussed in popular open platforms with the help of a mixed-methods approach. Reddit posts yielded 12,136 comments, YouTube videos 14,515, and TikTok videos 17,059, respectively. Out of these posts/entries, most represented matches related to sleep-related issues, including insomnia (n = 620 matches); anxiety (n = 353); depression (n = 204); and mental health issues in general (n = 165). After the initiation of GLP-1 RAs, losing weight was associated with either a marked improvement or, in some cases, a deterioration, in mood; increase/decrease in anxiety/insomnia; and better control of a range of addictive behaviors. The challenges of accessing these medications were a hot topic as well. To the best of our knowledge, this is the first study documenting if and how GLP-1 RAs are perceived as affecting mood, mental health, and behaviors. Establishing a clear cause-and-effect link between metabolic diseases, depression and medications is difficult because of their possible reciprocal relationship, shared underlying mechanisms and individual differences. Further research is needed to better understand the safety profile of these molecules and their putative impact on behavioral and non-behavioral addictions.

## 1. Introduction

Depression, diabetes, and obesity are potentially interrelated [1,2,3,4] and represent major public and individual health threats. These conditions have a negative impact on people’s quality of life and are associated with serious medical complications [5,6,7]. Their treatment still represents an ongoing challenge for clinicians. Recently, new drugs belonging to the GLP-1 receptor agonists (GLP-1 RA) class to treat metabolic diseases such as obesity and type 2 diabetes [8,9,10,11] have been made available. Given their proven clinical efficacy in being associated with weight loss, which for some of these molecules is similar to bariatric surgery [12], GLP-1 RAs immediately aroused great interest [13,14,15]. Indeed, several celebrities have promoted their effects, contributing to a major boost in their popularity so that Ozempic and GLP-1 RAs have recently been the object of pervasive interest on social media as well as in prestigious academic and non-academic venues [16,17]. This, in turn, has recently been associated with significant problems related to their distribution and availability through conventional channels and regular prescription methods [18,19]. However, whilst the welcome for GLP-1 RA as weight loss agents has been enthusiastic, there have also been criticisms and concerns [20,21,22,23]. Indeed, recent reports emphasized liraglutide and semaglutide GLP-1 RA-associated risk of triggering depression, suicidal thoughts, and self-injury [24]. In the case of semaglutide, this specific warning had already been made explicit for Wegovy but not for Ozempic [25,26,27]. Although a number of issues are still unclear [28], EMA recently decided to closely monitor the issue, especially with certain GLP-1 RA formulations [24].

Previously, weight loss agents such as rimonabant [29] have been withdrawn from the market due to suicidal issues. A vast range of medications [30], including antidepressants [31,32,33,34], have been associated with both depression and suicidal ideation. In addition to individual vulnerability levels in developing a specific adverse drug reaction (ADR), one could argue that significant levels of media attention to an index issue may increase the levels of ADR reporting. Overall, however, for a molecule to be included in the Food and Drug Administration (FDA) Black Box Warning labelling, significant levels of evidence need to be made available [35]. 

Consistent with the above, and in line with previous social media-based work [36,37,38,39,40,41], we aimed to assess the possible impact of GLP-1 agonists on mental health in general as being perceived and discussed in popular open web platforms [42]. In carrying out these quantitative and qualitative analyses, recent advances in the use of both artificial intelligence (AI) and natural language processing were considered as well.

## 2. Materials and Methods

Data were collected from Reddit, TikTok and YouTube [43,44,45]; their related content was freely available from the open web. These ‘snapshot’ data gathering activities were carried out on 28 May 2023, and further updated on 13 June 2023, with the help of specialized web application (e.g., Exportcomments) [46]. The web application retrospectively collected all entries made since the inception of the different subreddits examined here, which were created over a timeframe spanning from December 2019 to November 2022. Most social media users’ comments, however, were actually made during the Spring of 2023. The following keywords were used here: Ozempic, semaglutide, tirzepatide, Mounjaro, Wegovy and Rybelsus. Other keywords were also included in the research (i.e., dulaglutide, liraglutide, albiglutide, exenatide, lixisenatide, Trulicity, Victoza and Saxenda), but there were few related comments and hence considered irrelevant for the current analysis. Collected raw data were imported into Word and Microsoft Excel spreadsheets (Microsoft Office Professional 2021, Version 2308 Build 16731.20170). A range of selected keywords of interest (e.g., depression, mood, mental health, anxiety, compulsion, panic, sleep, and insomnia) were then searched within the archived whole set of comments. The resulting comments containing the same keyword(s) were then grouped together and analysed using specialized software [47]. Comments were initially sampled in groups of 10, and the following input was used to analyse each group of comments [=ai(“ I want you to act as an expert in qualitative content analysis and analyse these posts for me. identify all the themes and then present them in bullet points. please also consider any potential biases or contextual factors that may impact your analysis”)]. The whole set of themes and biases relating to each keyword generated by Numerous.ai (e.g., the n = 204 depression-related comments were divided in 21 groups) was then analysed by using ChatGPT 3.5 using the following input “could you identify the 5 most frequent themes/biases among these?”. An unobtrusive, mixed-methods, qualitative and quantitative, netnographic approach [40,48] was then adopted. For data assessment and interpretation, a phenomenological qualitative media analysis [4] was undertaken as well. In line with similar studies [49,50], for each keyword selected here, the most common resulting themes were then analysed manually. 

### Confidentiality and Ethics

In line with previous studies [40,41], only publicly available data from the selected social media were analysed; no access to any private or protected accounts was undertaken. Full anonymity was guaranteed, and no aliases/references to the Reddit/TikTok/YouTube users were collected/analysed. Confidentiality measures applied to the dataset included storage in an online, password-protected computer and removal of screen pseudonyms, URLs, country, and city identifiers.

Ethical approval for the study was granted by the Department of Pharmacy Ethics Committee at the University of Hertfordshire (protocol number: aLMS/SF/UH/02951(4)).

## 3. Results

A large range of comments were pulled from Reddit, TikTok, and YouTube. The keywords “*ozempic*”, “*semaglutide*”, “*tirzepatide*”, “*mounjaro*”, “*wegovy*” and “*rybelsus*” attracted the most attention and were analysed. With posts being an individual element within a thread, Reddit posts yielded 12,136 comments, YouTube videos 14,515, and TikTok videos 17,059, respectively. Some 5859 threads were also identified as a potential source for extracting additional comments. Reddit threads were extracted from the following subreddits: *r/Ozempic*, *r/OzempicForWeightLoss*, *r/Semaglutide*, *r/Tirzepatide*, *r/WegovyWeightLoss* and *r/Mounjaro*. Table 1 reports the number of matches from all comments for the searched keywords: “*depression*”, “*mood*”, “*mental health*”, “*anxiety*”, “*panic*”, “*compulsion*”, “*sleep*/*insomnia*”. A qualitative analysis was then performed using an assisted AI methodology (see above), which led to the identification of the five most common themes for each keyword (Table 2).

As can be seen from Table 2, mental health discussions relating to weight loss, GLP-1 RAs and obesity covered a wide range of topics, highlighting the complex and interconnected nature of mental health and weight management. In being analysed separately, the above themes showed a tendency to provide overlapping results; hence, a few areas will be here discussed in more detail, and examples of related, illustrative posts will be provided as well. 

### 3.1. The Complex Interrelation between Weight and Overall Levels of Psychological Wellbeing

One of the main issues discussed here was the interrelation between excessive weight and feelings of worthlessness, guilt, and stigma. According to a range of posts, obesity leads to the worsening of existing mental health problems or the development of new ones. Conversely, losing weight was associated with either a marked improvement or a deterioration of the mood itself.

- Positive reports

*e.g., This medicine is a game changer for people battling obesity. Obesity is so much more than being fat. It’s dealing with infections, joint pain, asthma, depression, and so many other comorbidities that sometimes make it feel impossible to live a more active lifestyle (…) This drug will literally be a life saver for many obese people.*

*e.g., I really think that this shot triggered changes in the habits in me that I had not noticed building up over years, habits that was using as crutches. Being able to “detox” off alcohol, anxiety and medication without feeling like the world was ending too? I think that resulted in getting pregnant.*

*e.g., My mental health has improved dramatically. I find myself feeling weird about feeling so normal and not hiding in my bedroom from the world. I actually want to discuss lower doses or stopping some of my medications for my anxiety, depression and PTSD with my doctor. My normal has been just turn it off in my head and live in the shadows.*

*e.g., (…) There’s medical research showing it has other mechanisms of effect on your brains rewards systems and dopamine receptors. Personally, I’ve had lifelong adhd, depression, and anxiety, and I’ve never felt like a medication completely worked. Vyvanse came close, so did adderall, but only while they were in my system and it was still touch and go. Semaglutide on the other hand felt like what people SAY antidepressants are supposed to feel like.*

*e.g., I have not had this problem. I have anxiety and depression and both have stayed stable since starting Weygovy.*

- Negative reports

*e.g., I was on wegovy for a month. Within a few weeks, I was so depressed, I wasnt leaving my room, or spending time with my kids. I did the bare minimum. I have had depression before, but weeks of being solitary has never been my experience. I havent lost weight either. I dont know if its from the drug or not, but I am skipping my shot today. I now have seen that depression is a side affect, along with extreme fatigue (…).*

*e.g., This IMMEDIATELY threw my hormones and emotions into a downward spiral. I’m infuriated that they don’t list anxiety and depression as an official side effect yet it’s all over the internet. I actually became angry for no apparent reason within 1 h of taking this witch potion and felt miserable for days until it wore off. Never again.*

*e.g., My now ex takes this. In under six months he lost 150 pounds. He did not inform his doctor that he suffers from anxiety, depression, and is bipolar. The drug should not be taken if you have any of those. His rages, delusions, and erratic behaviour dramatically increased, to the point that he was a completely different person. Christmas Day he destroyed my office and attacked me. Please, be very cautious about taking this drug. It is dangerous.*

*e.g., At this point, I don’t think I can psychologically continue with Ozempic. It’s making me afraid of solid food; I can’t meet my work, relationship, and home responsibilities; and I feel too awful to exercise or enjoy life. Going to the ER sucks. I think I’m done for good (…).*

*e.g., Yep it gave me the worst anxiety and depression. I had no choice to get off it after 5 kg weight loss only. Life was not worth living at that point.*

*e.g., Please remember that Ozempic can cause suicidal thoughts. … Anxiety, Panic Attacks, Suicidal thoughts, etc. Not all people will experience this side effect, but please remember that if you do feel you are depressed, more anxious, etc., please check your medication. Ozempic has taken many lives and it is NOT commonly known that it can cause these issues. PLEASE do not take this medication if you feel this could compromise your mental health just for the sake of ‘weight loss’.*

### 3.2. Weight Loss Medication Intake and Either Occurrence, or Improvement, of: Sleep Disturbances; Anxiety; “Food Noise”; Suicidal Ideation; Addictive Behavior

A range of posts were identified commenting on the appearance, or the amelioration, of a range of mental health-specific disturbances, including the achievement of an unexpected control over a range of addictive behaviors comorbid with obesity after the initiation of GLP-1 analogues.

- Sleep-related issues

*e.g., anyone noticed that tirz has affected their sleep? i have a really hard time falling asleep and when i sleep i usually toss and turn and wake up over and over, then wake up in the morning feeling literally hungover. i took my first dose of tirz on monday afternoon (2.5 mg) and i swear to god i have slept like a baby every night since. waking up feeling refreshed, rested, and so on. it’s been amazing!*

*e.g., My energy levels have skyrocketed, I no longer need a midday nap, have a better sleep schedule since I’m not waking up feeling like I’m starving in the middle of the night. This also may have to do with my weight loss and diet changes.*

*e.g., Has anyone else been restless before bed or have insomnia? I’m about 6 weeks in and noticed this entire last week and a half that I’ve been restless in my legs and up, just up, and I want to sleep but can’t. Just curious if this is happening to anyone else?*

*e.g., So I posted a question about insomnia etc. from tirz. I like literally have slept only a couple hrs since starting it. Would Ozempic have the same situation? Anyone have experience w this? Thanks!*

- Anxiety issues

*e.g., Jan 1 and went to 0.5. got crazy anxiety. after 3 weeks went back down to btwn .25-.5. this past week went up to .5. no side effects this time really.*

*e.g., The first month was full of side effects and brought on severe chest pains- called the paramedics and the EKG confirmed no heart attack, my follow up with the doctor also confirmed i was fine, blood pressure is good, resting heart rate was higher but still in a good range, and based on a mental health screening, they believe it was a panic attack—which 100% makes sense based on my history.*

*e.g., … Severe anxiety, constant panic attacks, insomnia, hypoglycaemia* 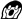

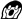

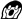
.

- “Food noise”

*e.g., That’s what’s amazing about these medications. It does quiet the food compulsion noises and desires to binge eat. The food noise just goes away. The 4 weeks I had access to the medication was amazingly silent in my compulsion to eat my feelings away.*

*e.g., One of the best things about Ozempic for me is that it quiets the food noise in my brain. By which I mean that Ozempic frees up the mental and emotional energy I used to spend on either thinking about food or thinking about not eating food I very much want to scarf down.*

- Addictive behavior

*e.g., It has been fascinating to watch people come into this sub and talk about how Sema has curbed their cravings for alcohol/cigs/online shopping. I think these drugs are potentially even more powerful than we originally thought. They have tremendous potential to be a force for good in the world—freeing people from their maladapted compulsions and anxiety.*

*e.g., My binging or impulsive tendencies has ceased. Coffee, tea, Dr. Pepper and online shopping are not happening.*

*e.g., (…) I have personally noticed not getting the “reward” from shopping, alcohol, etc*. 

### 3.3. Weight Loss Drugs; Related Safety and Challenges in Medications’ Access Issues

Quite a number of users discussed the somatic side effects of GLP-1 RA, typically including nausea, vomiting, diarrhoea. Very popular here were also comments on the challenges (e.g., insurance restrictions, high costs, and lack of enough stocks of molecules being made available) of accessing these medications.

*e.g., Has anyone experienced this? I’m 39 years old female, I do hit the gym hard when I can but it’s difficult. I’ve noticed especially on my main lifts my heart rate sky rockers to 170–195 and I get a little scared. I try to rest in between to bring it back down but it’s causing ….feeling extremely weak/dizzy, etc.*

*e.g., I was prescribed Ozempic a year ago for weight loss. I was 180 lbs and lost 30 lbs and when I got off of it I gained it all back do to anxiety and depression. It was impossible for me to continue eating portions that small when the side effects wore off (…).*

*e.g., I’m trying to find a reason why I feel so shitty. I think Ozempic decrease the absorption of the meds I take orally. I take a lot of meds that could be considered stimulating, Adderall for ADHD and Wellbutrin for depression. Since I started taking the Ozempic it feels like I’m not taking those two meds at all, I feel exhausted, unmotivated, and unfocused all day.*

*e.g., I am considering compounded because I don’t have insurance coverage (…) and even though I got a Wegovy prescription and would pay out-of-pocket with the coupon for at least a month to see what happens it seems to be out of stock everywhere I’ve looked. (…).*

*e.g., It wasn’t just social media and celebrities ramping up demand. My husband’s doctor prescribed it to him even though he doesn’t have diabetes and suggested it unprompted. …. Insurance shot it down, and I’m glad it did. Doctor did NOT mention mental health risks of it to my husband even though he was going to the doctor because of mental health issues. The media is partially to blame, but don’t pretend that doctors weren’t also pushing it to too many of their patients unnecessarily in order to get pharmaceutical kickbacks.*

*e.g., I was on Ozempic Dec-April for T2D and my insurance changed their requirements and isn’t covering it anymore. My last dose was the first week of April and the last two weeks have been horrible for me. … I’m feeling so defeated and asked my doctors office to appeal insurance’s decision, but who knows how long that will take and if they’ll allow me to take it again.*

*e.g., This is so heartbreaking that people are using a diabetic medication for weight loss. This isn’t a trend this is stealing medication from people who need it. It’s disgusting. Exercise, eat right and get good sleep. Stop stealing medications for major diseases.*

*e.g., At the beginning of this year, I weighed 351 pounds … As of today, March 19, I am already down to 318. And that’s with the gradual stepping up of the dose. I have the full support of everyone in my life, including my diabetic wife, who has personally experienced not being to get her Ozempic due to people using it for what I call “trendy weight loss”.*

*e.g., i have growth hormone and thyroid hormone deficiency, which makes it extremely hard to lose weight due to a extremely slow metabolism. i’ve been on wegovy for a month now, and i’ve already lost 6–7 lbs. i’m so happy that i am able to get this medication, as it has been drastically improving my vitals (bp, etc.) and my mental health. i feel awful to anyone that is unable to get the medication due to how much it’s being prescribed.*

*e.g., My endocrinologist prescribed mounjaro to me in September and I’m on the og SC. I am 5′1 and was at my highest weight of 267. Prediabetic, IR, sleep apnea, anxiety/depression, hypothyroid, and Hashimotos. Felt horrible, joints hurt, out of breath. Ask me about any gym, exercise program, bariatric doctors, medications, etc. and I have tried them all and could tell you about them. Some would work but would always gain back double to the point that nothing worked anymore. Mounjaro in a sense, saved my life. I’m 201 and finally feeling human again. However, here are my woes…every PA and appeal and peer review has been denied because BCBS doesn’t cover it without type 2 diabetes. Wegovy with their SC is still $1700 for me. I need something to help with binge eating and food noise and to help my body know how to work correctly. After June, I’m at a loss.*

## 4. Discussion

To the best of our knowledge, this is the very first paper aimed at assessing, with the help of qualitative and quantitative/mixed-methods [51,52], social media listening/netnographic [53,54,55,56,57] approaches, the GLP-1 receptor agonists’ possible related mental health issues as being perceived and described by a large number of open web and social media platform (e.g., YouTube, TikTok, Reddit) customers. The focus here was on the most represented topics and discussions that were identified, in line with recent literature studies [58,59,60], with the help of both Artificial Intelligence and natural language processing.

Previous similar studies aimed at describing social media entries/posts relating to anti-obesity drugs’ adherence, efficacy, and side effects [61]; analysing online trends of GLP-1 RAs [13,62]; or describing diabetics’ online behavior using netnography [63]. Social media listening and content analysis have been previously used to investigate the experiences and drug use patterns of individuals from a wide range of clinical and potentially harmful conditions, such as drug misuse, antidepressant use, obesity and depression [61,64,65,66,67,68,69,70,71]. These methodological approaches have also been employed to examine potential new clinical trends during specific time periods (e.g., the COVID-19 pandemic) [40,72,73].

Overall, it is relevant to note that current findings, mostly relating to customers’ observations made in the first part of 2023, commented in real time, and somehow possibly anticipated as well, a range of concerns reported by both media and clinicians during the last few months of 2023 [42,74,75]. This observation may not be surprising; indeed, social media have become an important part of our lives, influencing the way we spend time. In recent years, social media are increasingly used as resources and sources of information [76,77,78]. Current findings seem to confirm that these platforms are extensively used to share a huge and varied range of information. However, the huge social media interest observed here may have been associated with both a recent GLP-1 RAs drug shortage and a range of improper self-administration reports in non-clinically obese, non-diabetic, non-prescribed subjects [18,25,79]. Overall, users showed an intense need to compare their experiences with their peers, especially so to discuss specific symptoms, similar to what happens with recreational drug trip reports [40,80,81,82]. Conversely, this comparison-seeking behavior, in the case of prescribed medications, may be associated with the need to access more pharmaceutical- and diabetes/obesity-related data. From this point of view, however, recent literature has highlighted the potential risks of both an infodemic of false or misleading information [83,84] and a syndemic, which not only complicates the understanding of clinical conditions but also their resolution through an exacerbation of the public health problem [85]. 

Another issue that emerged here was the strong sense of “*self-efficacy*”, the “*joy*” of losing weight and the perceived sense of control over the amount of ingested food. A large number of both posts and pictures highlighted here the “*before and after*” effects of treatment, suggesting a form of pride that drove users to share such results online. Some seemed to consider weight reduction as a more important goal than health effects. The number of kilograms/pounds lost was a common topic of discussion, along with apprehension about regaining one’s original weight after discontinuation of treatment due to lack of medication, side effects or prescribing reasons. From this point of view, semaglutide and other GLP-1 RAs could be considered to some extent as Image and Performance Enhancing Drugs (IPEDs) [42]. 

People with diabetes and obesity show levels of comorbidity with depression [1]. Managing type 2 diabetes, including taking medicines, maintaining a healthy lifestyle, and monitoring blood glucose levels can be stressful, and this can be a factor in the development of depression and thoughts of suicide. Furthermore, the combination of type 2 diabetes and depression may be particularly evident in deprived socio-economic groups, possibly due to food insecurity issues [1,86,87,88,89]. Based on the content of current posts, these inequalities were possibly behind those posts reporting difficulties in both getting a prescription and communicating with physicians, which, in turn, may be due to healthcare access and health insurance issues. 

GLP-1 RAs have been considered antidepressants [90,91], either in monotherapy or in combination [92]. Indeed, GLP-1 RA-related “rapid” lifestyle changes due to significant/dramatic weight loss [12] may have a positive effect on the individual. However, GLP-1 RAs are reportedly involved in the regulation of both inflammation [91,93] and hormones involved in mood and appetite regulation, such as cortisol and thyroid hormones [94]. These issues may further explain GLP-1 RAs’ antidepressant effects. 

Conversely, analysing the different range of posts, a variety of descriptions suggesting the occurrence of a GLP-1 RA-related depression emerged here. One could argue that this was possibly associated with pre-existing mental health conditions. Conversely, a range of platform users described the potentially negative impacts of GLP-1 RAs’ related hypoglycaemic states on their mood. Of note, insulin-induced hypoglycemia is a common cause of emergency department calls [95], and abnormal glycaemic homeostasis has been related to the onset of mental illness [96]. Type 2 diabetes, especially in patients with depressive symptoms, is associated with increasing levels of suicidal behavior and suicidal ideation; among the risk factors, there is unsatisfactory glycaemic control [97,98,99,100]. Furthermore, body mass index has a role in suicidality, and suicidal thoughts are still under investigation in obesity [101,102,103]. Whilst GLP-1 RAs improve glycaemic control by increasing insulin secretion and decreasing glucagon production [104,105], fluctuations in blood glucose levels may influence mood and may increase the risk of developing depressive symptoms. It is also worth noting that several users feared a relapse of depressive symptoms after discontinuation of treatment. 

Depression may manifest itself in some obese subjects not only with lowering of mood but also with anxiety, insomnia, appetite changes [106], impulsivity [107,108], and rumination issues/“*food noise*” [109]. In line with these observations, previous studies showed that although GLP-1 RAs may present with antidepressant effects [87,91], they may possess *anxiogenic* effects [110,111]. In fact, the GLP-1 RA-related potential modification of GABAergic neurotransmission [112,113] may help in explaining the occurrence of complex symptom constellations such as anxiety, sleep disorders, and relief from depression. 

Finally, the putative effect of GLP-1 RAs on craving in relation to behavioral and non-behavioral addictions was fairly extensively commented on by social platform users. An overall impact on cravings in GLP-1 RA-prescribed subjects has been discussed [114,115,116,117]. Indeed, the brain’s dopaminergic reward system is involved in motivation, mood, pleasure, and food cravings [118,119]. From this point of view, one could argue that the satiety effects relating to GLP-1 RAs, resulting in clear weight loss effects, may well be related to their direct/indirect action on the dopamine reward system. 

### Limitations

Data utilization from social platforms comes with limitations. Indeed, different from other data collection methods, such as focus groups or interviews, on social media people, tend to speak freely, and that by itself is an advantage. Social media data analysis may, however, imply biases, methodological pitfalls and ethical boundaries. From this point of view, strict adherence to ethical guidelines is essential since this ensures user anonymity and appropriate data use, although it may inherently limit the depth of personal user data analysis. The lack of experimental control complicates the determination of causality, and data integrity can suffer from users’ inaccuracies and omissions. Researcher bias, linguistic heterogeneity and self-presentation tendencies increase the burden of data rigour. Furthermore, a more granular, platform-specific analysis could have helped here in achieving better levels of understanding of social media users’ comments. A further bias was here given by the sole use of English as the language chosen for the analysis. Again, whilst the selected keywords efficiently directed the data extraction, the same choice of keywords inherently may have been associated with a certain level of selection bias, and, as a result, some relevant discussions might have been missed. The broader applicability of findings is influenced by platform demographics, while temporal and spatial variables can affect data representativeness. More specifically, while the study has described user-generated perceptions, it may also inadvertently have captured reactions to external events that influenced these platforms’ users during the data collection period chosen here. A further important issue is the identity of those writing on social networking sites. Some may present with anonymity; others may use different nicknames; a few may engage in trolling behavior; and finally, an individual may possess several identities/aliases/usernames and post the same material. Furthermore, social media, including Reddit, users are only partially representative of people who use drugs in general since not all drug misusers have access to computers and/or smartphones.

The current study uncovered a variety of biases strictly related to online studies and data collection methodology, e.g., selection bias due to the fora’s focus on weight loss drugs, confirmation bias driven by users’ personal experiences, psychological traits, socio-cultural and economic biases influencing beliefs. Media, context, cultural and social influence also contribute. While Reddit, TikTok, and YouTube were chosen for their popularity and reach, user demographics and characteristics may well differ across platforms. Thus, current findings might not be wholly representative of other online platforms, broader offline perceptions, and the whole community of people using GLP-1 RAs, a population that may include no-fixed-abode subjects, such as individuals from deprived areas, etc. Moreover, differences between the GLP1-RAs’ routes of administration were not analysed in detail, nor was a comparison performed between the individual molecules.

To cope, however, with the possible inadequacy of a dataset based on a range of self-reporting statements, with the current cherry-picking approach [120,121], a range of interesting results was still identified and discussed, somewhat supporting the validity of the large sample of posts/comments analysed here. 

The use of artificial intelligence (AI) and natural language processing may be another reason for methodological debate. The novel AI-based approach allowed a fast and effective qualitative analysis, reducing the operator time and bias. Overall, these approaches have enabled new techniques for analysing textual data, attracting research interest in various fields, e.g., social sciences, drug discovery, and marketing [122,123,124,125,126]. Generative AI’s multifaced role has arisen as a significant frontier in augmenting cognitive processes and driving innovation in scientific investigation. This technology offers a new approach to imagination and intellectual exploration by redefining both thinking and creativity. AI chatbots, including ChatGPT, seem to be valuable aids for scientists, assisting in material organization and facilitating data elaboration. As well, research shows that AI text analysis carries the risk of propagating biases and errors from training datasets due to its reliance on machine learning from such data. The lack of transparency in complex neural networks aggravates this concern by hindering the ability to provide clear explanations and accountability [127,128,129]. However, a cautious use with constant human supervision, as here carried out, to avoid the well-known and typical AI’s “*hallucinations*” [130,131,132,133,134], can be helpful in mixed and integrated approaches, for example, to facilitate the analysis of vast quantities of textual data.

## 5. Conclusions

It appears from the results reported here that some novel antidiabetics were perceived as affecting mood and behavior. A range of factors may influence, independently, depression and mental health in patients taking GLP-1 RAs, such as pre-existing risk factors for depression or suicidal ideation, for example, a family history of mental illness or recent stressful events, and co-occurring medications. Establishing a clear cause-and-effect link between metabolic diseases, depression and medications is difficult because of their possible reciprocal relationship, shared underlying mechanisms and individual differences. Further research is needed to better understand both these complex interactions; the possible clinical impact of GLP-1 RAs on behavioral and non-behavioral addictions; and the safety profile [135] of these molecules. Finally, one issue looming at the door, still largely ignored in social media and public discourse, is that despite the current hype, GLP-1 RAs may be losing effects over time and rebound mechanisms in terms of weight loss and behavioral outcomes should be expected, as it has been here suggested by a few social media users. Hence, it is conceivable that a second wave of comments from disappointed users will soon occupy the blogosphere.

## Figures and Tables

**Table 1:** Selected keywords and number of matches from all extracted comments.

Keywords	Number of Matches from All Comments
Depression Mood Mental health Anxiety Panic Compulsion Sleep disorders/insomnia	204 62 165 353 66 19 620

**Table 2:** The 5 most common themes for each keyword.

**Mental Health**
**Themes**
Weight loss and gain, including struggles with obesity and efforts to lose weight through various means like diet, exercise, and medication.
Health conditions associated with obesity and weight gain, such as metabolic syndrome, diabetes, high blood pressure, and elevated cholesterol levels, as well as mental health conditions that contribute to weight gain.
Medication, specifically the use of GLP-1/GIP injectables like Ozempic and Wegovy for weight loss, including discussions about their efficacy and side effects.
Mental health and its role in weight loss efforts, as well as the impact of weight on mental health, including issues of self-worth, guilt, and stigma.
Access to healthcare and medication, with discussions about insurance restrictions, high costs, and challenges in obtaining weight loss medications.
**Depression**
**Themes**
Personal experiences with weight loss and using Ozempic as a treatment.
Potential side effects of Ozempic, including insomnia and its impact on mental health (depression, anxiety).
The relationship between Ozempic and depression/anxiety, as well as concerns about the safety and potential negative effects of the medication, especially for those with mental health conditions.
The impact of mental health (depression, anxiety, OCD, etc.) on weight, weight loss struggles, and the use of medications for treatment.
Criticisms of weight loss methods, pharmaceutical drugs, and the healthcare industry as a whole, including issues with insurance coverage, medication interactions, and a focus on profit-driven motives.
**Mood**
**Themes**
Medication for Weight Loss: Discussions about the effectiveness and side effects of various weight loss medications, including both positive and negative experiences with different drugs.
Diet and Lifestyle: Conversations about specific diets like keto and processed foods, as well as the importance of exercise and lifestyle changes for weight loss.
Mood Changes: Users sharing experiences of mood changes associated with medications, both positive and negative, as well as mood improvements after weight loss.
Medication Side effects: Discussions about various physical side effects experienced by users while taking weight loss medications, such as stomach cramps, reduced hunger, and changes in menstrual cycles.
Hormonal and Mental Health: Users discussing the impact of weight loss medications on hormones, mental health, mood stabilization, and their effects on conditions like PMDD, ADHD, PTSD, and depression.
**Anxiety**
**Themes**
Positive impact of weight loss on mental health: Many users mention the positive effects of weight loss on mental health, particularly improvements in anxiety and depression.
Side effects of medication: Several posts discuss the various side effects experienced while taking weight loss medications, such as nausea, anxiety, insomnia, and others.
Personal experiences with medication: Users share their individual experiences with weight loss medications, both positive and negative, including their efficacy and impact on mental well-being.
Anxiety and mental health struggles: Anxiety is a recurring theme, with users expressing concerns about anxiety related to weight loss, medication, and other health conditions.
Struggles with weight loss and health conditions: Some users discuss their challenges in managing weight loss, dealing with other health conditions, and the impact on their mental health.
**Compulsion**
**Themes**
Compulsion: Many users discuss their struggles with compulsive behaviors, such as eating, shopping, and hair pulling.
Medication: Users extensively discuss the effectiveness of medications like Ozempic and semaglutide in controlling their compulsion and reducing cravings.
Weight loss: Users express skepticism about using medication solely for weight loss and emphasize the importance of addressing the underlying causes of their compulsion.
Positive effects: Many users report positive effects beyond weight loss, including increased energy, clearer thinking, and reduced anxiety from managing their compulsion.
Other compulsions: Users share their success in controlling other compulsive behaviors like smoking and shopping.
**Sleep**
**Themes**
Side effects and experiences with medication for weight loss: Users discuss various medications like Ozempic, Wegovy, semaglutide, and Mounjaro and their impact on weight loss, appetite, and overall health. Side effects such as nausea, fatigue, sleep disturbances, and digestive issues are frequently mentioned.
Sleep issues and their relation to weight loss and medication: Many users share their struggles with sleep, including insomnia, disrupted sleep patterns, and the impact of medication on sleep quality. Some also discuss the potential benefits of improved sleep on weight loss efforts.
Weight loss challenges and personal struggles: Users talk about their individual weight loss journeys, including challenges they face, efforts to lose weight through exercise, diet, or medication, and concerns about slow progress or potential barriers.
Health conditions and their impact on weight and weight loss: Various health conditions like PCOS, diabetes, hypertension, sleep apnea, and thyroid issues are discussed in relation to weight management and the effectiveness of different weight loss methods.
Lifestyle changes and alternative weight loss methods: Conversations mention lifestyle changes, including exercise, diet adjustments, fasting, and other self-control strategies, as alternative methods for weight loss before considering medication.
**Insomnia**
**Themes**
Insomnia: Insomnia appears to be a significant concern, being mentioned multiple times in different contexts, such as related to Ozempic usage, fasting, and medication changes. Users discuss experiencing difficulty sleeping and restlessness before bedtime, and they mention the potential impact of Ozempic on causing insomnia.
Ozempic and its side effects: There is a focus on Ozempic, primarily in relation to weight loss, but also as a potential cause of insomnia and other side effects. Users mention experiencing dry mouth, mood swings, headaches, stomachaches, heartburn, and other normal aches and pains while using Ozempic.
Weight gain and weight loss: Weight-related topics are frequently discussed, particularly in the context of Ozempic’s potential for weight loss and users’ concerns about weight gain.
Medication changes and interactions: Users talk about making changes to their medications, including prescriptions for insomnia and anti-depressants. Additionally, there are concerns about the impact of Ozempic on other medications they may be taking.
Appetite suppression and food-related issues: Some users mention experiencing appetite suppression as a result of Ozempic usage, while others talk about self-soothing with food or food cravings in the context of insomnia and mental health improvements.

## Data Availability

The data that support the findings of this study are available from the corresponding author upon reasonable request.
